# ‘Heat-Treatment Aqueous Two Phase System’ for Purification of Serine Protease from Kesinai (*Streblus asper*) Leaves

**DOI:** 10.3390/molecules161210202

**Published:** 2011-12-08

**Authors:** Amid Mehrnoush, Shuhaimi Mustafa, Abdul Manap Mohd Yazid

**Affiliations:** 1 Department of Food Technology, Faculty of Food Science and Technology, Universiti Putra Malaysia, 43400 UPM Serdang, Selangor, Malaysia; Email: Mehrnoush_amid@yahoo.com; 2 Department of Microbiology, Faculty of Biotechnology and Biomolecular Science, Universiti Putra Malaysia, 43400 UPM Serdang, Selangor, Malaysia; Email: Shuhaimi@biotech.upm.edu.my

**Keywords:** purification, heat treatment, aqueous two phase system, Kesinai, serine protease, yield

## Abstract

A ‘Heat treatment aqueous two phase system’ was employed for the first time to purify serine protease from kesinai (*Streblus asper*) leaves. In this study, introduction of heat treatment procedure in serine protease purification was investigated. In addition, the effects of different molecular weights of polyethylene glycol (PEG 4000, 6000 and 8000) at concentrations of 8, 16 and 21% (w/w) as well as salts (Na-citrate, MgSO_4_ and K_2_HPO_4_) at concentrations of 12, 15, 18% (w/w) on serine protease partition behavior were studied. Optimum conditions for serine protease purification were achieved in the PEG-rich phase with composition of 16% PEG6000-15% MgSO_4_. Also, thermal treatment of kesinai leaves at 55 °C for 15 min resulted in higher purity and recovery yield compared to the non-heat treatment sample. Furthermore, this study investigated the effects of various concentrations of NaCl addition (2, 4, 6 and 8% w/w) and different pH (4, 7 and 9) on the optimization of the system to obtain high yields of the enzyme. The recovery of serine protease was significantly enhanced in the presence of 4% (w/w) of NaCl at pH 7.0. Based on this system, the purification factor was increased 14.4 fold and achieved a high yield of 96.7%.

## 1. Introduction

Proteolytic enzymes are a class of proteins found extensively in animals and plants as well as in microorganisms because of their ubiquitous nature [1]. Plant serine proteases are important for their functional properties in many physiological process, e.g., microsorogenies, transduction and signal differentiation, hypersensitivity responses and degradation of proteins [2]. The high proteolytic activity of plant serine protease over wide temperature and pH ranges, and also its stability towards various surfactants and bleaching agents, have meant that plant serine protease is suitable for use in the food, medicinal, biotechnology and pharmacology industries [3]. Kesinai (*Streblus asper*) is a plant found in tropical and subtropical regions [4]. It has been reported that various parts of the kesinai have medicinal properties such as antigingivitis, antidote for snake bites, antidysentery and in wound healing [5]. In addition, it has been reported that the leaves of kesinai are a rich source of proteolytic enzymes and thus a potential material for protease purification [6].

However, the wide applications of the enzyme in medicine and biotechnology require a quick and easy purification method which can lead to improved overall yield and purity. Furthermore, scaling up this process should be simple and a continuous steady state if possible [7]. Conventionally, methods like homogenization, centrifugation, filtration, dialysis and precipitation are used for the purification of serine protease from plants to obtain crude feedstock. Chromatography is then employed for purification of the enzymes [8]. An attractive technique for the production of industrial enzymes compared to the conventional method of purification is the ‘heat treatment aqueous two-phase system’ (‘heat treatment-ATPS’) integrating concentration, clarification and initial purification of protein directly from crude feedstock. Furthermore, heat treatment of feedstock, a relatively cheap and simple method, has been successfully used as a selective preliminary purification step in order to reduce the level of feedstock contaminants. Currently, there is no information regarding the purification of serine protease from plant using ‘heat treatment-ATPS’. Therefore, the effect of heat treatment and other important parameters such as molecular weight of polymer, salt, pH and the addition of NaCl on the recovery of serine protease from kesinai (*Streblus asper*) leaves and on purification factors were investigated in this study.

## 2. Results and Discussions

### 2.1. Effect of Temperature on Purity of Serine Protease from Kesinai Leaves

**Figure 1:**
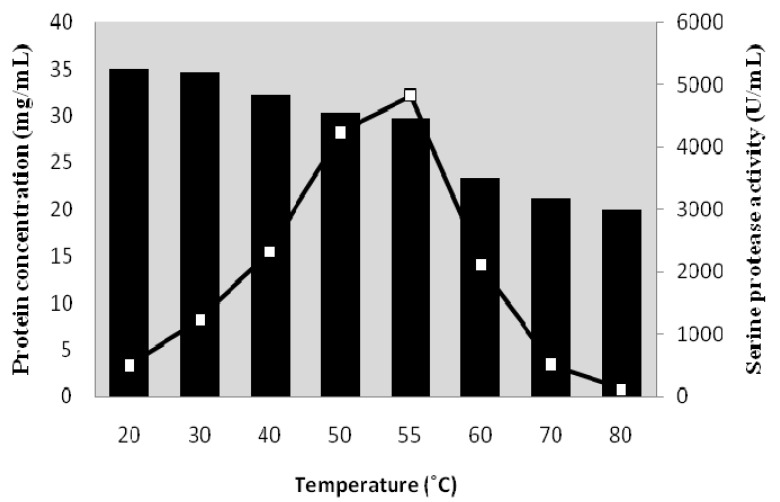
Effect of heat-treatment on serine protease activity (□) and protein concentration (█) of enzyme. The residual serine protease activity was determined after incubation of the enzyme at various temperatures (20 to 80 °C) and various interval times (5–60 min).

**Figure 2:**
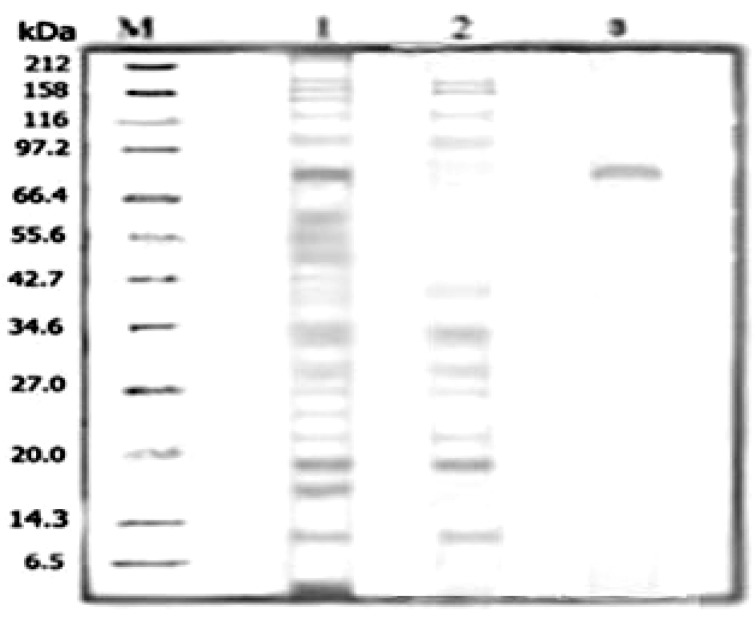
The purity of the partitioned serine protease was assessed by a 12% SDS-PAGE analysis. Samples from top phase were procured from the PEG/Salt ATPS with a composition of 16% (w/w) PEG, 15% MgSO4 and 4% (w/v) NaCl. M = protein molecular markers (6.5–212kDa); Lane 1 = non-heat treatment crude feedstock; Lane 2 = heat treatment crude feedstock; Lane 3 = Purified serine protease in top phase.

The effect of different temperatures on serine protease activity and protein concentration of heat-treated crude enzyme was studied to determine the best temperature and duration of thermal treatment in the purification method. Optimization of heat-treatment of crude enzyme showed that the highest serine protease activity and lowest protein concentration was achieved at 55 °C after 20 min (Figure 1). The highest enzyme activity and lowest protein concentration of the heat-treated sample implied that temperature reduces the level of contaminating proteins. As shown in Figure 2, the heat-treated crude enzyme indicates lesser and fainter bands (lane 2) than non-heat treatment crude enzyme (lane 1) due to denaturation and precipitation of undesirable protein. Therefore, SDS-PAGE analysis confirms that heat-treatment of the crude enzyme could improve the purity of target enzyme. Therefore, based on this result, the crude enzyme was heated at 55 °C for 20 min before using in the purification system in future experiments.

### 2.2. SDS-PAGE Analysis of the Purified Serine Protease

The composition of 16% PEG6000-15% MgSO_4_, 4% (w/w) NaCl addition at pH 7.0 was selected for SDS-PAGE analysis of purified serine protease using heat-treatment ATPS. The purity of the enzyme was evaluated by 12% SDS-PAGE. Figure 1 shows the SDS_PAGE profile of the standard protein markers, heat treatment, and non-heat treatment crude extracts and purified enzyme. As shown in the figure, the non-heat treatment crude feedstock contains a range of bands (lane 1), representing impurities present in the feedstock. As considered earlier, the heat-treated crude feedstock indicates the lesser bands in order to reduce the level of contaminant proteins (lane 2). The bottom phase also shows the lesser bound compared to the non-heat treatment crude extract (lane 4), while, the sample collected from the top phase with highest proteolytic activity shows one dark band (lane 3) with molecular mass 75.2 kDa. Therefore, this SDS-PAGE result shows that the purification technique employed in this study gives a maximal recovery of serine protease from kesinai leaves.

### 2.3. Effect of PEG on the Partitioning of Serine Protease from Kesinai Leaves

The influence of different PEG molecular weights (4,000–8,000 g/mol) and different concentrations (8–21%, w/w) on protein partition coefficient (K_P_) and enzyme partition coefficient (K_E_) was investigated. As shown in Table 1, the partitioning of serine protease from kesinai leaves is strongly dependent on PEG molecular weight and concentration. The distribution of protein and protease in two phases was reported in ‘heat treatment ATPS’ by K_P_ and K_E_, respectively.

The high values of Kp and K_E_ indicate that more protein and target enzyme respectively were partitioned to the top phase [9]. Based on the results, the K_E_ significantly decreased at low concentration and molecular weight of PEG (Table 1).This was possibly because in this condition the concentration and molecular weight of PEG was not suitable for sufficient partitioning and decreased the presence of the target enzyme in the upper phase [10].

**Table 1:** The effect of PEG concentration and molecular weight on partitioning of serine protease.

Phase Composition (%, w/w)	K_P_	K_E_	PF	Yield (%)
8% PEG4000-15% MgSO_4_	ns	ns	ns	ns
16% PEG4000-15% MgSO_4_	0.24 ± 0.03	1.4 ± 0.09	4.4 ± 0.13	41.3 ± 0.22
21% PEG4000-15% MgSO_4_	0.50 ± 0.10	0.03 ± 0.01	3.2 ± 0.10	33.8 ± 0.31
8% PEG6000-15% MgSO_4_	0.22 ± 0.74	2.12 ± 0.02	6.2 ± 0.8	53.4 ± 0.13
16% PEG6000-15% MgSO_4_	0.001 ± 0.23	4.52 ± 0.04	8.9 ± 0.02	83.1 ± 0.08
21% PEG6000-15% MgSO_4_	0.04 ± 0.18	3.51 ± 0.14	7.8 ± 0.2	61 ± 0.06
8% PEG8000-15% MgSO_4_	0.31 ± 0.15	1.02 ± 0.12	5.3 ± 0.03	50.2 ± 0.12
16% PEG8000-15% MgSO_4_	0.07 ± 0.07	0.23 ± 0.01	3.9 ± 0.01	31.9 ± 0.03
21% PEG8000-15% MgSO_4_	0.10 ± 0.02	0.31 ± 0.21	4.2 ± 0.05	40.2 ± 0.05

^Kp^: partition coefficient of protein in top phase; ^KE^: partition coefficient of enzyme in top phase; ^PF^: Purification factor of enzyme; ^Yield^: activity recovery; ^ns^: no phase separation.

On the other hand, the high PEG concentration and molecular weight showed a negative effect on partition coefficient of the enzyme (K_E_). Under such conditions, the concentration of target enzyme in the bottom phase was increased due to the increase in the exclusion volume effect of PEG over salting out effect of salt [11]. Also, the purification factor and yield were decreased at high PEG concentration and molecular weight; this could be because there was more target protein than other proteins in the bottom phase. From the results obtained, the intermediate molecular mass and concentration of PEG showed the best value for partitioning of serine protease to the top phase with high purification factor (8.9) and yield (83.1%). Therefore, the PEG6000 at 16% was selected to study the effects of salts on partitioning of protease.

### 2.4. Effect of Salts on the Partitioning of Serine Protease from Kesinai Leaves

One of the important parameters to improve the phase partitioning of target biomolecules between two phases in ATPS is salt [12]. In general, phase separation is not observed in the presence of PEG or salt alone. This shows that the combination of PEG and salt is necessary to achieve phase separation. Thus, the different types of salts (Na-citrate, MgSO_4_ and K_2_HPO_4_) with various concentrations were prepared to determine their effect on partitioning of serine protease. The results did not show any phase separation at 12% (w/w) of sodium citrate, magnesium sulfate and potassium phosphate (Table 2). It could be because the salt concentration required for two-phase formation in partitioning of serine protease was inadequate.

**Table 2:** Effect of phase composition in 16% PEG6000-salts on partitioning of serine protease.

Phase Composition (%,w/w)	K_P_	K_E_	PF	Yield (%)
16% PEG6000-12% Na-citrate	ns	ns	ns	ns
16% PEG6000-15% Na-citrate	0.12 ± 0.02	1.8 ± 0.15	3.8 ± 0.80	34 ± 0.09
16% PEG6000-18% Na-citrate	0.24 ± 0.17	0.8 ± 0.40	1.6 ± 0.17	21 ± 0.07
16% PEG6000-12% MgSO_4_	0.12 ± 0.23	4.1 ± 0.31	7.4 ± 0.14	74 ± 0.07
16% PEG6000-15% MgSO_4_	0.02 ± 0.01	5.38 ± 0.06	11.3 ± 0.21	87.2 ± 0.4
16% PEG6000-18% MgSO_4_	0.08 ± 0.11	3.4 ± 0.21	18.5 ± 0.31	62 ± 0.82
16% PEG6000-12% K_2_HPO_4_	0.31 ± 0.02	2.9 ± 0.21	5.1 ± 0.04	52 ± 0.10
16% PEG6000-15% K_2_HPO_4_	0.23 ± 0.18	3.8 ± 0.32	6.8 ± 0.22	63 ± 0.02
16% PEG6000-18% K_2_HPO_4_	0.18 ± 0.04	3.1 ± 0.11	4.3 ± 0.02	48 ± 0.12

^Kp^: partition coefficient of protein in top phase; ^KE^:partition coefficient of enzyme in top phase; ^PF^: Purification factor of enzyme; ^Yield^: activity recovery; ^ns^: no phase separation.

As shown in Table 2, the partitioning of enzyme was significantly influenced by the type and concentration of salts. There was a decrease in partition parameters when the concentration of salts was increased to 18% (w/w). It can be seen that the enhancement of salt increased the salting out effect, with both contaminated protein and target enzyme being more partitioned to the top phase and leading to decreased purification factor and yield of the enzyme (Table 2). Another reason for the shift of target enzyme and non-desired protein to the top phase with increased salt levels was probably due to the similar hydrophobic properties on their surfaces [13].

It has been reported that the efficiency of salts for phase partitioning can be described by a lyotropic series (a classification of ions based upon salting-out or salting-in ability) [14]. The nature of the ions indicates their effectiveness in salting-out or salting-in. It has been found that the multi-charged anions are the most effective in partitioning (SO_4_^−2^ > HPO_4_^−2^ > acetate) compared to cations [(NH_4_^+^) > K^+^ > Na^+^ > Mg^2+^ > Ca^+2^] [15]. As shown in Table 2, at a concentration of (15%, w/w) of salt, SO_4_^−2^ had more effect on purification factor and yield compared to HPO_4_^−2^ and acetate, respectively. Therefore, partitioning of serine protease at a salt concentration of 15% (w/w) is influenced by anions rather than cations. Based on the results, the highest serine protease purification factor (11.3) and yield (87.2%) was obtained at 16% PEG6000-15% MgSO_4_. Thus, this composition was chosen to investigate the effect of pH on partitioning of the enzyme.

### 2.5. Effect of pH on the Partitioning of Serine Protease from Kesinai Leaves

**Figure 3:**
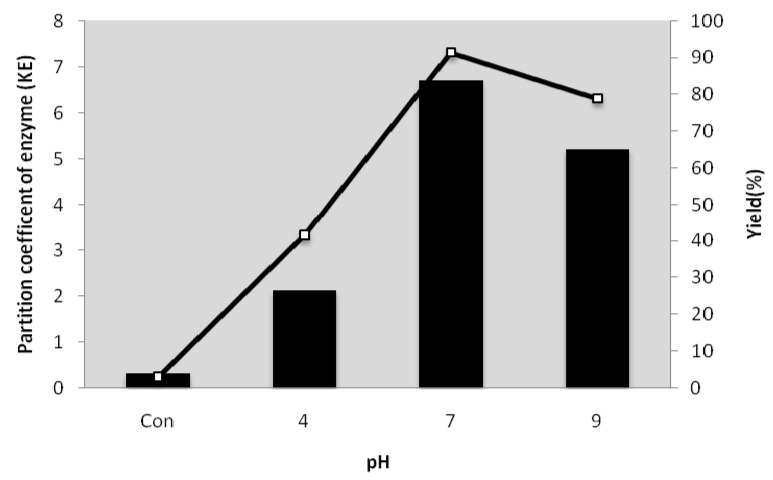
The pH of ‘heat treatment ATPS’ was varied between 4.0 and 9.0. The serine protease partition coefficient (█) and yield (□) were calculated using Equations 2 and 5 accordingly.

The heat-treatment ATPS with a composition of 16% PEG6000-15% MgSO_4_ was employed to investigate the effect of pH on partitioning of serine protease from kesinai leaves. The pH of the system was adjusted to pH 4, 7 and 9 compared to the pH 6 of the control system. The K_E_ and protease recovery were increased in the pH range from 7.0 to 9.0 and the highest serine protease yield (91.3%) and enzyme partition coefficient (6.7) were achieved at pH 7.0 (Figure 3). In general, the pH affects the partitioning of protein by changing the charge of the solution or by altering the charge ratio of the protein [16]. Negatively charged protein tends to act with PEG and positively charged protein is partitioned to the bottom salt phase [17]. Therefore, the interaction of the protein and PEG becomes stronger above the isoelectric point (*pI*) because the protein gets negatively charged under this condition and the partition coefficient increases.

Based on this explanation, the K_E_ and recovery of the serine protease were decreased at pH 4 because at this pH the enzyme was positively charged and preferred the salt-rich phase. On the other hand, when the charge of protein was changed from positive charge to neutral, the partition coefficient and yield of the enzyme were significantly increased [18].This could be the reason for the increase of partition coefficient and recovery of serine protease at pH 7.0 and 9.0 because the proteins were close to their natural charge. The partition coefficient and recovery of serine protease were low at pH 9.0 compared to pH 7.0 and this could be due to fact the active site of the enzyme was in an inappropriate state at alkaline pH.

### 2.6. Effect of NaCl on the Partitioning of Serine Protease from Kesinai Leaves

The influence of NaCl on partitioning of serine protease was investigated with the composition of 16% PEG6000-15% MgSO_4_.The water structure and hydrophobic interactions can be differently affected by the addition of NaCl in ATPS [19], where the interaction between hydrophobic chain (ethylene group) of PEG and hydrophobic surface area of the enzyme was enhanced. Abbott and Hatton [20] have reported that NaCl affects yield by inducing the phase separation or by changing electrical potential.

**Figure 4:**
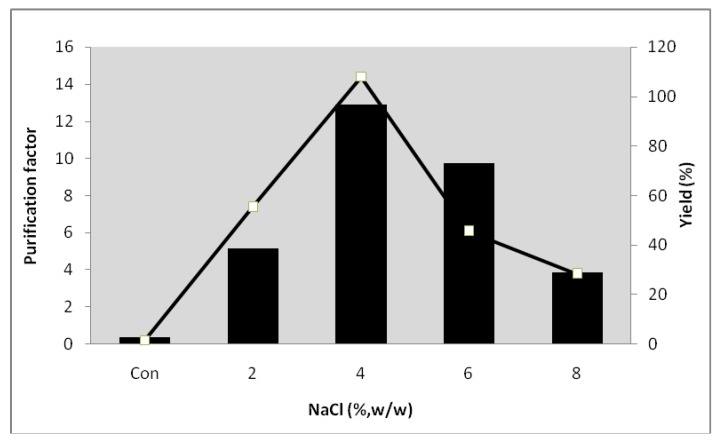
All the scouting experiments were carried out with 16% PEG 6000-15% MgSO_4_ at pH 7.0. The purification factor (□) and yield (█) were calculated as a function of the NaCl concentration.

It was also reported by Marcos *et al*. [21] that the partitioning of penicillin acylase was significantly increased by the addition of NaCl to PEG-salt based ATPS. Chaiwut *et al*. [9] revealed that the recovery of protease from *Calotropis procera* latex was markedly unhanced when NaCl was added to the system. Based on the result, the highest purification factor (14.4) and yield (96.7%) of serine protease were achieved with the addition of 4% (w/w) of NaCl (Figure 4). The addition of NaCl above 4% (w/w) caused a decrease in the recovery of the enzyme due to the unequal partitioning of natural salts between two phases thus affecting the chemical potential of the solute.

## 3. Experimental

### 3.1. Plant Materials

The kesinai leaves (*Streblus asper*) were obtained from several kesinai plants available at Universiti Putra Malaysia, (Selangor, Malaysia) Science Park. The leaves were immediately washed with distilled water and kept at 4 °C for 1 h prior extraction.

### 3.2. Chemicals

All chemicals and reagents used were analytical grade. Pectin from citrus fruits, bovine serum albumin (BSA) and Bradford reagent were supplied by Sigma Chemical Co., (St. Louis, MO, USA). Polyethylene glycol (PEG), Sodium dodeycel sulfate (SDS), trichloroacetic acid (TCA, 99%), di-sodium hydrogen anhydrous, sodium hydrogen phosphate monohydrate, di-potassium hydrogen phosphate and potassium di-hydrogen phosphate, were purchased from Merck (Darmstadt, Germany).

### 3.3. Enzyme Extraction

Twenty grams of fresh leaves were washed with double-distilled water, then blended with 100 mM sodium phosphate hydrogen at pH 7.5 (40 mL) in a Waring commercial blender 32BL79 (Torrington, CT, USA) at high speed for 4 min. The homogenate was filtered through cheesecloth and the filtrate was centrifuged at 8,000 rpm for 20 min at 4 °C to produce crude enzyme [6].

### 3.4. Optimization of Heat-Treatment on Crude Enzyme

The crude enzyme was heat-treated in a different range of temperatures (20–80 °C) for 60 min to determine the best temperature. Following this, another set of crude enzyme was heat-treated at different interval times (5–60 min) to determine the best treatment duration. Finally, residual serine protease activity and protein concentration of each set was determined based on the methods used by Whooley *et al*. [22] and Costa *et al*. [23].

### 3.5. Preparation of ‘Heat Treatment Aqueous Two Phase System’

This involved the heat treatment with ATPS prepared in 15 mL centrifuge tubes. Different concentrations and PEG molecular mass (4,000–8,000) as well as salts (Na-citrate, MgSO_4_ and K_2_HPO_4_) were added to 20% (w/w) of heat-treated crude enzyme from kesinai leaves. Subsequently, a sufficient amount of distilled water was added to the system to achieve a final mass of 10 g. Gentle agitation was done for equilibration and then centrifugation (4,000 × *g* for 15 min) was used to achieve a phase separation. Removal of the upper phase was carried out with the aid of a pipette and the lower phase was afterwards collected. To minimize interferences of PEG and salts the controls of each system were prepared by the addition of 20% (w/w) distilled water instead of enzyme. The purity of the system was determined by protease activity assay, protein concentration determination, and SDS polyacrylamide gel analysis.

### 3.6. Effect of PEG on the Partitioning of Serine Protease

To investigate the effect of PEG on serine protease partitioning, various molecular weights of PEG (4,000, 6,000 and 8,000 g/mol) at different concentrations of the polymer (8, 16 and 21%, w/w) were mixed with 15% (w/w) of MgSo_4_ in the system. The serine protease activity and protein concentration of top and bottom phases were determined. The phase composition, which gave the highest serine protease yield, was selected for further study.

### 3.7. Effects of pH and NaCl on Partitioning of Serine Protease

The composition of PEG and salt with the highest serine protease yield was chosen to determine the optimum pH and NaCl for partitioning of serine protease. This was for the purpose of investigating the effects of different pH (4, 7 and 9) and different concentrations of NaCl (0, 2, 4, 6 and 8%, w/w) on the partitioning of the enzyme. Separation of phase was achieved by centrifugation after the whole system was filled up to 10 g.

### 3.8. Analytical Methods

#### 3.8.1. Serine Protease Activity Assay

An enzyme sample of 0.1 mL was added to 1 mL of 0.2% (w/v) azo-casein in 100 mM sodium phosphate hydrogen at pH 7.5. The mixture was incubated in a water-bath at 60 °C for 20 min thus producing a proteolytic reaction which was stopped by adding 0.5 mL of 30% TCA to the mixture. This was followed by centrifugation at 13,400 rpm for 10 min and the supernatant was obtained. The serine protease activity in the supernatant was measured with a spectrophotometer at 335 nm [22] and the results expressed as a mean of three readings with an estimated error of ±10%.

#### 3.8.2. Protein Concentration Determination

The protein concentration of enzyme was determined by the method described by Costa *et al*. [23]; BSA was used as standard.

#### 3.8.3. Determination of Partition Coefficient, Specific Activity, Purification Factor and Yield

The partition coefficient of protein (K_P_) was determined by taking the ratio of protein concentration in the top phase (P_T_) and dividing it by protein concentration in the bottom phase (P_B_) (Equation 1):

K_P_ = *P_T_*/*P_B_* (1)

The partition coefficient of enzyme (K_A_) was defined as:

K_A_ = *A_T_/A_B_* (2)

where A_T_ and A_B_ are activities of the enzyme in the top and bottom phases respectively.

The specific activity of the enzyme was determined by taking the ratio of total activity of serine protease and dividing it by serine protease total protein (Equation 3)

Specific activity (*U/mg*) = *Total activity (U)/Total protein (mg)* (3)

The purification factor of enzyme in the top phase (P_FT_) was measured by the specific activity of the serine protease in the top phase divided by the initial specific activity of the enzyme (Equation 4):

P_FT_ = *Specific Activity of Top Phase Sample* / *Specific Activity of Crude Feedstock* (4)

The yield (Y_T_) of serine protease was defined using Equation 5:

Y_T_ (%) = *100*/*1* + *(1/[V_R_]. [K_e_])* (5)

where K_e_ is the partition coefficient of serine protease and V_R_ is the volume ratio of the top phase to the bottom phase [24].

#### 3.8.4. Sodium Dodecyl Sulfate-polyacrylamide Gel Electrophoresis (SDS-PAGE)

SDS-PAGE of the protein was done according to Laemmli [25] in electrophoresis unit (Bio-Rad) using an acrylamide gel consisting of 12% resolving gel and 4.5% stacking gel. A 10% tricholoacetic acid (TCA) solution was used to concentrate and precipitate protein samples that had been removed from the top phase. The resuspension of the pellets was achieved with a sample buffer .The process of electrophoresis was used at 110 V and 36 mA for 60 min. Protein bands were observed using the silver staining method [26].

## 4. Conclusions

This study demonstrates that the heat-treatment procedure by precipitation of undesirable proteins could enhance the purity of serine protease obtained from kesinai leaves. The composition of 16% PEG6000-15% MgSO_4_ system with 4% NaCl addition at pH 7.0 recovers serine protease from kesinai leaves with high purification factor (14.4) and yield (96.7%). It is also evident that the molecular mass and concentration of PEG and salt as well as NaCl and pH have significant effect on partitioning of the enzyme. Adding salts and modifying the pH improves the partitioning of serine protease into the top phase, Furthermore, the finding indicates an alternative process option for effective and cheap purification of serine protease, which is possible using heat-treatment ATPS. Hence, this method could also be employed in large scale purification of enzyme from crude feedstock.
